# Cardiac Patients’ Experiences and Perceptions of Social Media: Mixed-Methods Study

**DOI:** 10.2196/jmir.8081

**Published:** 2017-09-15

**Authors:** Stephanie R Partridge, Anne C Grunseit, Patrick Gallagher, Becky Freeman, Blythe J O'Hara, Lis Neubeck, Sarah Due, Glenn Paull, Ding Ding, Adrian Bauman, Philayrath Phongsavan, Kellie Roach, Leonie Sadler, Helen Glinatsis, Robyn Gallagher

**Affiliations:** ^1^ Prevention Research Collaboration Sydney School of Public Health, Charles Perkins Centre The University of Sydney Sydney Australia; ^2^ School of Health and Social Care Edinburgh Napier University Edinburgh United Kingdom; ^3^ Sydney Nursing School Charles Perkins Centre University of Sydney Sydney Australia; ^4^ School of Nursing and Midwifery Faculty of Medicine, Nursing, and Health Sciences Flinders University Adelaide Australia; ^5^ St. George Hospital South Eastern Sydney Local Health District Sydney Australia; ^6^ Ryde Hospital Northern Sydney Local Health District Sydney Australia; ^7^ Manly Hospital Northern Sydney Local Health District Sydney Australia; ^8^ Royal North Shore Hospital Northern Sydney Local Health District Sydney Australia

**Keywords:** cardiovascular disease, cardiac rehabilitation, qualitative research, secondary prevention, social media

## Abstract

**Background:**

Traditional in-person cardiac rehabilitation has substantial benefits for cardiac patients, which are offset by poor attendance. The rapid increase in social media use in older adults provides an opportunity to reach patients who are eligible for cardiac rehabilitation but unable to attend traditional face-to-face groups. However, there is a paucity of research on cardiac patients’ experiences and perspectives on using social media to support their health.

**Objective:**

The aim of this study was to describe cardiac rehabilitation patients’ experiences in using social media in general and their perspective on using social media, particularly Facebook, to support their cardiac health and secondary prevention efforts.

**Methods:**

A mixed-methods study was undertaken among cardiac rehabilitation patients in both urban and rural areas. First, this study included a survey (n=284) on social media use and capability. Second, six focus group interviews were conducted with current Facebook users (n=18) to elucidate Facebook experience and perspectives.

**Results:**

Social media use was low (28.0%, 79/282) but more common in participants who were under 70 years of age, employed, and had completed high school. Social media users accessed Web-based information on general health issues (65%, 51/79), medications (56%, 44/79), and heart health (43%, 34/79). Participants were motivated to invest time in using Facebook for “keeping in touch” with family and friends and to be informed by expert cardiac health professionals and fellow cardiac participants if given the opportunity. It appeared that participants who had a higher level of Facebook capability (understanding of features and the consequences of their use and efficiency in use) spent more time on Facebook and reported higher levels of “liking,” commenting, or sharing posts. Furthermore, higher Facebook capability appeared to increase a participants’ willingness to participate in a cardiac Facebook support group. More capable users were more receptive to the use of Facebook for cardiac rehabilitation and more likely to express interest in providing peer support. Recommended features for a cardiac rehabilitation Facebook group included a closed group, expert cardiac professional involvement, provision of cardiac health information, and ensuring trustworthiness of the group.

**Conclusions:**

Cardiac health professionals have an opportunity to capitalize on cardiac patients’ motivations and social media, mostly Facebook, as well as the capability for supporting cardiac rehabilitation and secondary prevention. Participants’ favored purposeful time spent on Facebook and their cardiac health provides such a purpose for a Facebook intervention. The study results will inform the development of a Facebook intervention for secondary prevention of cardiovascular disease.

## Introduction

Coronary heart disease (CHD) is a leading cause of death worldwide [1]. A sizeable proportion of the burden of CHD is avoidable by targeting modifiable risk factors [2] such as tobacco use, high blood pressure, physical inactivity, poor diet, high body mass, and alcohol use [2]. Furthermore, attendance at group-based cardiac rehabilitation programs improves risk factors and subsequently reduces recurrent events [3]. Despite the benefits of cardiac rehabilitation, a vast majority of eligible patients, particularly those aged over 65 years, do not attend in-person services [4,5] because of geographical barriers, misconceptions, scheduling of programs, and conflicting demands [6,7].

Rapid growth in the use of the Internet and mobile phones, including smartphones, has emerged as a potential solution to improve access to cardiac rehabilitation. In 2016, 87% and 68% of adult populations from countries with advanced economies reported using the Internet and owning a smartphone, respectively [8]. These rates are even higher among the Australian adult population, with 93% using the Internet and 77% owning a smartphone [8]. Even among adults aged 65 years or older, the use of mobile phones and social media are becoming deeply embedded in everyday life [9]. Pervasive adoption of technology offers alternative opportunities for researchers and clinicians to engage with this hard-to-reach cardiac population [4,5] and establish services to meet the needs of future generations.

Platforms, such as Facebook, which enable social interactions through user-generated content, are collectively known as social media [10]. Social media usage among adults aged over 65 years has more than tripled from 2010 to 2015, from 11% to 35% in the United States [11]. Similarly, in Australia (2012-13), among the 46% of adults aged over 65 years who use the Internet, social media use is one of the four top Web-based activities [12]. As social influences are a primary factor in the adoption of health behaviors [13,14] and a core component of cardiac rehabilitation [15], adapting social support elements from cardiac rehabilitation programs for adjunct delivery via social media platforms is a logical progression to ensure flexible delivery and to maximize program reach [16].

The most commonly used platform for adults aged over 65 years in Australia [17] and the United States [9] is Facebook. As a result of the increase in Facebook popularity among this population, social support elements for electronic health (eHealth) or mobile health (mHealth) cardiac rehabilitation programs are often integrated into investigator-designed platforms as an adjunct to a multicomponent program [18,19]. However, these programs often do not detail the social media component or the role of user experience in their development. Understanding the user experience of Facebook and the potential to use this platform to achieve behavior change is essential to ensure the effectiveness of any program developed [20,21]. To date, however, there is only limited research on patient acceptance of existing social media platforms, such as Facebook, as a potential delivery modality for cardiac rehabilitation.

This study aims to describe cardiac rehabilitation patients’ social media use; and among current users, their experience and perspectives on using Facebook to support their cardiac health through secondary prevention.

## Methods

### Design and Participants

This is a two phase mixed-methods study. Phase 1 comprised a survey of technology use in participants eligible for or currently attending cardiac rehabilitation. This survey was a component of a larger study (n=282) that screened cardiac patients regarding many aspects of their overall health technology use. Phase 2 comprised a series of focus groups with cardiac rehabilitation participants who were current Facebook users to elucidate their Facebook experience and the potential for using Facebook for the delivery and support of cardiac rehabilitation.

Phase 1 participants were cardiac patients recruited from cardiac hospital wards as inpatients or outpatients attending cardiac rehabilitation programs in New South Wales (NSW) at two metropolitan (7 hospitals) and three rural health districts (3 hospitals). Participants were candidates for the survey if they were eligible to be referred to cardiac rehabilitation and spoke and understood sufficient English for consent and questionnaire processes. Participants were excluded if they had a neurocognitive disorder or a major visual deficit.

Phase 2 participants were cardiac patients recruited from cardiac rehabilitation programs at one metropolitan and one rural hospital used in phase 1. Participants were eligible for this phase of the study, if in addition to the inclusion criteria for phase 1, they were currently using Facebook.

### Ethical Considerations

The studies were approved by Northern Sydney Local Health District Human Research Ethics Committee in March 2016 (HREC ref: LNR/15/HAWKE/450), and all patients gave written informed consent before participation.

### Data Collection

During phase 1, technology acceptance was assessed using a 20-item survey combining components of questionnaires developed by Edwards et al [22] and Illiger et al [23]. Questions included access to mobile technology, current usage patterns including features used, and confidence and interest in usage for health. Sociodemographic and clinical data were collected using a checklist developed for a previous study by the team [24]. This questionnaire was pilot-tested on 15 cardiac rehabilitation patients and modified to improve accuracy and specificity. Data collection for phase 1 took place from March to November 2016. This study reports only a subset of the data relating specifically to social media use in general.

During phase 2, the details of participants’ Facebook experiences were collected using focus group interviews (n=6). The focus groups were aimed at understanding participants’ Facebook experience, engagement, and confidence to identify factors associated with Facebook acceptance and to examine the potential for Facebook platforms to support their cardiac health and for secondary prevention options. A semistructured interview guide was used to elicit responses so that the question type and topics could vary according to participants’ answers, and issues raised by previous groups could be added. Each of the focus groups began with introductions and an outline of processes and aims from the facilitator (SRP). Thereafter, participants were encouraged to express their opinions and were provided with sufficient time to do so. Participants were also asked to complete a short questionnaire that included sociodemographic and clinical characteristics, as well as questions on access to a broad range of social media platforms, current usage patterns, and confidence and interest in using social media for health purposes. An additional researcher (PG or SD) was present throughout the interview to assist with organization and audio-recording and to write field notes. The focus group recordings were transcribed verbatim for analysis.

### Data Sources, Analysis, and Statistics

The survey data from phase 1 were analyzed using Statistical Package for the Social Sciences (SPSS) Statistics Version 22 (SPSS Inc). Descriptive statistics were used for continuous measures, including counts and percentages for participant demographic characteristics and technology use. Comparisons of demographic characteristics and technology use of non–social media users and social media users were conducted using chi-square tests.

The deidentified focus group transcripts were entered into NVivo version 10 software program (QSR International Pty Ltd). All focus groups were coded (SRP), allowing for data immersion and obtaining an overall sense of the data. An open coding approach was adopted, forming a general description of the research topic through generating codes and recurrent themes as they emerged. Discussion with additional researchers (AG and RG) familiar with the data finalized and confirmed emerging themes. Verbatim quotes illustrative of the themes and subthemes had been extracted. During this process of analyses, it became apparent that the participants’ preferences for features of a Facebook group suitable for cardiac rehabilitation attendees were being revealed. Therefore, a secondary analysis of the transcripts and field notes was undertaken to identify these aspects and preferences for current and future Facebook groups by one researcher (PG). Indications (both positive and negative) of the strength of preference for different aspects of social media groups were assessed in transcripts and field notes and independently assessed by another researcher (RG). The aspects and the associated negative and positive strengths of preference were discussed between 3 researchers (PG, SRP, and RG) until consensus was reached. Symbols are used to indicate the overall strength of preference, with “−−−” indicating the features considered to be least desirable and “+++” indicating those features considered most desirable.

## Results

### Phase 1: Technology Survey

#### Participant Profile

Survey results indicated that 28% (79/282) of the cardiac rehabilitation participants in the sample used social media regularly (Table 1). In comparison with nonusers, social media users were younger; more than half (38/75; 51%) of social media users were aged ≤59 years compared with 17% (33/202) of nonusers. A higher proportion of social media users compared with non–social media users were in full-time employment (47% [37/79] vs 23% [47/203], *P*<.001), had a secondary education qualification or higher (76% [60/79] vs 58% [118/203], *P*=.04), and regularly used smartphones (90% [71/79] vs 53% [108/203], *P*<.001) and tablets (64% [50/79] vs 34% [68/203], *P*<.001). Almost two-thirds of social media users compared with one-third of non–social media users were accessing information on health conditions (*P*<.001). Social media users compared with non–social media users were significantly more likely to use technology to access information on medications, heart conditions, heart treatments, and lifestyle changes. Additionally, 30% (24/79) of social media users compared with 12% (24/203) of nonusers were willing to communicate with health professionals using technology (*P*<.001). There was no statistically significant difference between social media users and nonusers with their willingness to use technology to communicate with other cardiac participants (8% [6/79] vs 4% [8/203], *P*=.17).

### Phase 2: Focus Groups

#### Participants’ Profile

Of the 20 participants invited to participate in the focus groups, 18 took part (72% urban). Focus group participants had a mean age of 63 years (standard deviation [SD] 12; range: 38-79 years). The majority were males (13/18), had some form of tertiary education (15/18; mean years of education=14.4, SD=2.7), and were white (13/18). The 5 non-white participants were of Indian, European, Aboriginal, or Pacific Islander descent. The majority of the participants were working part-time (4/18) or were retired (9/18) and were living with a partner (12/18). Among this group, one-third each had been admitted for myocardial infarctions (6/18), or percutaneous coronary interventions (6/18), or coronary artery bypass grafting or valve replacement surgery (5/18).

**Table 1:** Phase 1 survey: demographics and technology use by cardiac rehabilitation participants compared for social medial use (N=282).

Demographics and technology use			Non–social media users (N=203) n (%)	Social media users (N=79) n (%)	*P* value
**Demographic characteristics**					
	**Age in years**^a^**, n (%)**				
		49 or younger	6 (3.0)	11 (14)	<.001
		50-59	27 (13.4)	27 (36)	
		60-69	70 (34.7)	22 (29)	
		70-79	73 (36.1)	11 (14)	
		80 or older	26 (12.9)	4 (5)	
	**Gender, n (%)**				
		Female	57 (28.1)	21 (27)	.80
	**Language spoken at home, n (%)**				
		English	185 (91.1)	73 (92)	.55
	**Education level, n (%)**				
		≥year 12^b^	118 (58.1)	60 (76)	.004
	**Location, n (%)**				
		Metropolitan	144 (70.9)	69 (87)	.18
	**Employed, n (%)**				
		Full-time or part-time	47 (23.2)	37 (47)	<.001
**Technology use**					
	**Device, n (%)**				
		Tablet or iPad	68 (33.5)	50 (63)	<.001
		Smartphone	108 (53.2)	71 (90)	<.001
	**Accessing information, n (%)**				
		Health conditions	66 (32.5)	51 (65)	<.001
		Medications	54 (26.6)	44 (56)	<.001
		Heart condition	36 (17.7)	34 (43)	<.001
		Heart treatment	32 (15.8)	29 (37)	<.001
		Lifestyle changes	34 (16.7)	30 (38)	<.001
	**Communication, n (%)**				
		Health professionals	24 (11.8)	24 (30)	<.001
		Other cardiac patients	8 (3.9)	6 (8)	.17

^a^One non–social media user did not report age, and 4 social media users did not report age.

^b^Secondary education or higher.

**Figure 1:**
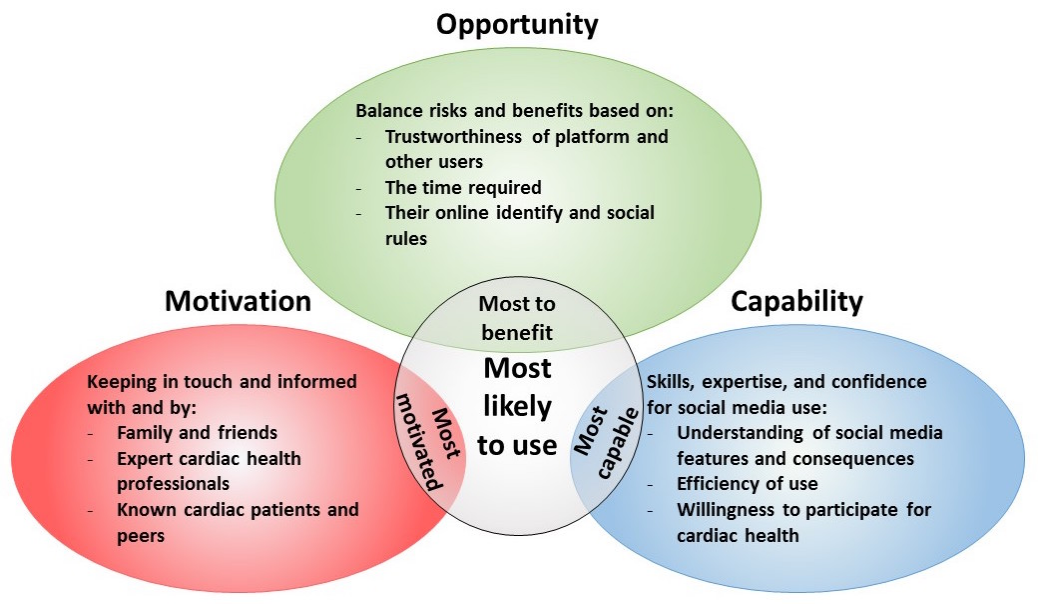
Social media use themes for cardiac rehabilitation patients.

The short questionnaire completed by participants before the start of the focus group found that the most commonly used social media site was Facebook (16/18), followed by Instagram (7/18), LinkedIn (5/18), and Twitter (5/18). The average number of social media sites per participants used was three (range: 1-5). The majority were multidevice users, with 15 of 18 using a computer or laptop, tablet, and a smartphone. Most participants accessed social media sites on their smartphone (13/18) and in combination with one other device—their computer or laptop (11/18) or their tablet (13/18).

#### Focus Group Themes

Participant discussion of their current Facebook use appeared to involve the interaction of three components, namely, motivation, opportunity, and apparent capability (Figure 1). As a result, the participants who were most likely to use Facebook demonstrated high levels of all three key attributes portrayed in Figure 1, that is, those most likely to use Facebook were also the most motivated, the most capable, and identified the most benefits from the potential opportunities Facebook provides. Participants were motivated to keep in touch with known family and friends, and they recognized that Facebook provided an opportunity for such social interactions. Participants’ use of Facebook strongly related to their apparent capability to use Facebook and their perceived benefits of social media use. For example, those perceiving fewer benefits of Facebook appeared or judged themselves to have lower Facebook capability and less motivation to use Facebook to keep in touch (ie, used other forms of communication). If given the opportunity to use a cardiac Facebook group, participants indicated they were motivated to do so to keep informed by expert health professionals, but again their willingness to participate was influenced by their Facebook capability.

#### Motivation

Participants described their main motivation for using Facebook generally was to “keep in touch” with family and friends, being informed, and informing each other of current life events. Facebook provided support for existing relationships as opposed to developing new relationships. For example:

I use it to keep in touch with friends, and groups, and uh, it’s very good for keeping touch with family that are living out interstate...It’s good. It helps you to keep in touch with people, finding out what they all know and what they’re doing.

Male, 69, focus group 1

The thing I like the most is it keeps me in contact, it lets me see what other people are doing which I normally wouldn’t do. Like, I’ve got lots of extended family and cousins and different things and so they’ll put things on about their kids or somebody’s gone on holidays and all that and I think that’s good because we’re all so busy nobody has time to make phone calls these days.

Female, 59, focus group 2

A secondary motivation for Facebook use described by younger participants as well as those who were older but “early adopters” was that it helped to fill in spare time. One of the participants stated:

The beauty of the phone is you can, you know, if you’re waiting for a train or waiting for anything you can entertain yourself as much or as little as you like.

Male, 69, focus group 1

However, the motivation to keep in touch with family and friends seemed to be the most powerful, influencing their development of skills to engage and the way they engaged with Facebook.

#### Opportunity

From the focus groups, it appeared that participants harness the opportunity Facebook provided to keep in touch with family and friends in different ways and carefully balanced risks and benefits in an ongoing way. For instance, participants weighed the opportunity to keep informed of family events against the risk (as they perceive it) of excessive advertisements. Similarly, the balance of the opportunity to engage in a conversation with family and friends was weighed against the time required to filter and make posts and the risk of generating unwelcome responses. Additionally, participants weighed up these stated benefits with the time required to develop and define their online identity and to understand the rules of social engagement on Facebook.

Most participants mentioned that their use of Facebook involved observing and sharing personal photos, “liking” photos or updates of family and friends, and communicating through private messaging features but seldom communicated through groups, as stated below:

I mostly browse but if it’s something that I really want to see, like I’ve got friends that live overseas and things like that and they’ll post cute photos of their kids and stuff, I definitely will like that and read it or something. Yeah, it’s a way of letting people know that you know what’s happening.

Female, 59, focus group 2

I use Facebook a lot and Facebook Messenger, which I like because it’s a private conversation and you don’t have all the other things that you have to scroll through.

Female, 79, focus group 1

Participants had varying levels of trust of both the Facebook platform and of other users. Trust was informed by other, non-Facebook (eg, email and Internet banking) Web-based experiences removed from Facebook platforms and impacted the way participants navigated and used the platform. Frequent, confident Facebook users reported developing strategies to deal with trust issues while still using the platform, which is echoed by the following:

...even though I’m on Facebook and Wi-Fi a lot, every single day, I would not share anything that I consider to be personal, private, financial, medical information through that media and never have. Because you can’t guarantee that it’s secure...but I’ve got to be very very sure that I’m using something that has a secure transaction.

Female, 58, focus group 3

I take it with a grain of salt. Facebook is one of those mediums where people can write whatever they want, there’s no factual evidence that supports what their saying, it’s just their opinion and I take it as that, as their opinion I never take it as complete fact.

Male, 38, focus group 3

Sometimes there’s too much on there and you just think “oh well, whatever I’ve missed I’ve missed I don’t have time.” But yeah I find...there [is] a lot other stuff on there that you don’t want to see.

Female, 59, focus group 2

However, less frequent or confident Facebook users did not describe having strategies in place to deal with their trust issues and reported less control of their online personal space. This increased their time on Facebook and appeared to negatively affect their Facebook experience, as expressed by some of the participants:

What I don’t like about Facebook is how you can say what you want, you can swear, every word known to man, now I’m not a prude and they get away with it; if you put that in the paper you’d have, you’d be sued. How do they allow that? That’s my question...I think it’s putrid, it’s filth. It’s putrid. But it’s technology.

Male, 73, focus group 6

It’s like 4 people come up you don’t know from a bar of soap wanting to be your friends, what’s all that?...I don’t know who they are.

Male, 59, focus group 6

Participants expressed the view that spending a large amount of time on Facebook was undesirable because of the potential negative perceptions by others. Increased time spent on Facebook is perceived to signal that the person has no other interests or they are oversharing personal information or personal opinions of current popular culture or current affairs, irrespective of trust and capability; this was reiterated by some of the participants:

I just think a person who’s posting a lot of stuff is spending a lot of time on Facebook...I think [they] should be doing something more with [their] life. That’s my personal opinion.

Female, 79, focus group 1

I use it a lot I guess, but I don’t like to sit on it all the time, I like to get on and off and just enjoy what I am doing and then if I am going to do something then I’ll go on it, because I can’t just stand sitting on the computer, there’s got to be a reason for being there.

Female, 58, focus group 5

However, it appears that participants would reach a point or threshold when they considered they had invested too much time, particularly when using handheld devices, and they then described developing strategies to reduce use. For example:

Actually, I just deleted [Facebook] on my phone because I got sick of...I’d look at it too much. So I decided I’m only going to look at it on my iPad, which is not with me all the time.

Female, 62, focus group 1

Participants’ online Facebook behaviors appear to be a reflection of the rules of behavior they consider acceptable in their social group and in relation to communication with their friends and family. This included developing strategies to tolerate specific family and friends and not changing their reactions for an online context, as noted by some participants:

You can unfollow and still be friends, I do that a lot. If someone keeps posting constantly you just click unfollow and you stay friends. They don’t know that you're not seeing it.

Female, 62, focus group 1

I wouldn’t say something on Facebook to anybody that I wouldn’t say to their face.

Male, 63, focus group 6

#### Capability

Participants’ engagement and perception of Facebook were influenced by their apparent capability. Comments by participants revealed that their capability of understanding and using Facebook fell on a continuum of skill, expertise, and confidence, which then influenced whether they used the opportunity Facebook provided.

Participants who were apparently less capable with regard to Facebook use revealed their lower level of understanding of Facebook features in distinct ways in their comments during the focus groups, such as being less able to control the number and type of posts and notifications they received. As a consequence, they were more likely to be distracted and annoyed by common Facebook features and the amount of perceived trivial communication. This also increased the amount of time they spent on Facebook to achieve what they wanted, thus perceiving it as a “necessary evil” (Male, 73, focus group 6). Oftentimes, less capable Facebook users were unable to distinguish between Facebook platforms and the wider Internet. Their sense of safety or risk was then falsely enhanced, extending beyond what is possible. For example, one of the participants noted:

I just got loaded with stuff and a lot of it was interesting, like I like bushwalking and I put down my interests. But then there was just so much going through that, I was bombarded, but I kept seeing things like “yes I’d like to do that one day” but in the end I just had to delete it, it was just overwhelming.

Female, 79, focus group 1

Participants who were apparently more capable revealed a better understanding of Facebook features and the consequences of their use through their easy navigation of content with limited distractions. They also described their communication through the confident use of multiple features:

If I’m just putting a post up myself or answering one of their posts then that would in the public domain and then when it came around to if we needed to discuss things of a family nature then that would go to private messaging.

Male, 56, focus group 4

If I’ve got something sensible to say I do, but a lot of people make silly comments and unnecessary comments and it is just time consuming.

Male, 69, focus group 1

Differences in participants’ apparent capability were also revealed through the language used to describe Facebook actions and the desire for simplicity. Some participants were aware of their lower level of capability in using Facebook and discussed that their lower skill level was a result of learning Facebook at an older age:

I mean, it’s [got to] be simple for our age group, I think. Like, that we don’t have to have other people to do it. You know, it’s [got to] be easy enough.

Female, 62, focus group 1

A lot of it is coming through the young people, the young ones with their work. They get very involved and learn about all these things, and we as the older generation learn from them. But it’s not something that we learned when we were at work so we’re kind of on the edge and catching up with the young ones. That’s what I find I do at times.

Female, 58, focus group 5

#### Potential Use of Facebook for Cardiac Rehabilitation

No participant raised the potential for using Facebook for their own health or specifically for cardiac health; however, after being prompted to consider this possibility, a lot of discussion ensued. Most participants discussed that Facebook, in general, would provide an opportunity for continued interaction with known expert cardiac health professionals and cardiac rehabilitation peers to receive further cardiac specific information and be provided with personalized cardiac information. Participants who were more capable Facebook users recognized the benefits of Facebook private groups for this purpose and were more willing to participate in a Facebook group and to help others through peer-to-peer learning, which is echoed by the following:

You know it is information at hand when you need it. You don’t have to physically go into a place and have a chat with someone, it’s there. Something that you use often and it’s available at a swipe. Someone can be there to answer any questions that you’ve got. I think it’s a great idea.

Male, 38, focus group 3

The beauty of it is it’s a very quick way of getting out information and it costs nothing, and it’s just very effective.

Male, 69, focus group 1

The other thing that may be a good idea either for myself or others is to be available to a group like that to mentor...because there are things that I’ve gone through, there are things that each of you, I'm sure had things that have been really beneficial, things that have been a struggle—to be a mentor to somebody, or a buddy, I don’t know call it what you will, that’s something I would be happy to do.

Female, 58, focus group 3

Participants with lower perceived benefits of Facebook and apparently lower capabilities were still willing to “*give it a go*” (Male, 79, focus group 5). Another participant noted:

I think that’s why people are cynical about Facebook...but if it is dealing with things that are, issues, whatever, then that’s a good thing. Like cardiac rehabilitation or something like that.

Male, 63, focus group 6

Some of the participants expressed their hesitation about the level of sharing of personal health information required and privacy and confidentiality of shared health information:

Yeah, I think it would do...be very good because you can share. Once you start sharing the tension and the unknown is not as great and that releases any stress that you would generally hold on and wonder what’s happening.

Female, 69, focus group 3

As long as there’s privacy and I could trust it and it could be guaranteed then yeah definitely. So there’s a trust issue there. So it couldn’t be accessed by people flogging products or services. My dealing is with you and the hospital and not with the mob that’s selling water bottles for example.

Male, 63, focus group 1

#### Recommendations of Features When Creating a Facebook Intervention for Cardiac Rehabilitation

In light of both the findings described above and the secondary analyses of preferences for Facebook, a series of recommendations have been generated regarding features to consider when creating a cardiac rehabilitation Facebook group (Table 2 **)**. Three categories need to be taken into consideration in relation to platform settings, the role of the moderator, and group interaction. Participants strongly preferred a closed or secret group, and they wanted to be able to access the platform on multiple devices. Strong preferences were also indicated for having an expert cardiac professional act as moderator and that content should focus on cardiac health information and provide link-outs to relevant material. Participants also emphasized the importance of trustworthiness of the group, whereas excessive notifications, advertisements, and hostile or augmentative posts were not favored.

**Table 2:** Facebook group preferences.

Categories and features			Preference^a^
**Platform settings**			
	“Closed” or “Secret” group	+++	
	Multiple device access	++	
	Presence of advertisements	−	
	Excessive notifications	−−	
**Moderator role**			
	Expert cardiac professional	+++	
	Cardiac health information	++	
	Cardiac information link-outs	++	
**Group interaction**			
	Trustworthiness of group	++	
	Ability to post or comment	+	
	Irrelevant posts	−	
	Hostile or argumentative posts	−−	

^a^+++ Most desirable; −−− Least desirable.

## Discussion

### Principal Findings

This mixed-methods study provides strong indications that a cardiac Facebook group has potential for cardiac rehabilitation purposes and provides a set of recommended features to consider when designing such a Facebook group. To our knowledge, this is the first study to elucidate cardiac rehabilitation participants’ perceptions of and engagement with social media and the potential for using Facebook for their cardiac health. Social media use was low but more common in participants who were under 70 years of age, employed, and had completed high school. It appeared cardiac rehabilitation participants’ Facebook engagement is influenced by a combination of personal motivation, opportunity, and capability of using the platform. When applied to cardiac rehabilitation, participants could be motivated to use Facebook because of the opportunity it provided to “keep in touch” with a known expert cardiac health professional and to further improve their cardiac health. More capable Facebook users may also be open to providing peer support in a social media group by acting as champions. The findings of this study will be used to develop a Facebook intervention for persons eligible for cardiac rehabilitation.

Key prerequisites in social media use in older adults were identified previously in a review by Leist [16] and include adoption of information and communications technology and social media–related knowledge, attitudes toward social media, and motivations for social media use. All participants in this study demonstrated varying levels of these prerequisites. A combination of their motivation to use Facebook for supporting their existing relationships and their capability of Facebook use seems to influence the participants’ Facebook-related knowledge and attitudes toward Facebook. Also similar to Leist [16], we found barriers such as lack of capability and skeptical attitudes that were described as more constraining with increasing age [25]. In this study, participants aged 70 years or older were much less likely to be using social media in general, which might limit the potential use of Facebook for cardiac rehabilitation. For instance, given that the mean age of heart attack participants in the study context of Australia is 67 years (SD 15), it is likely that many participants will not be capable of using a cardiac Facebook group option [26]. However, it is highly likely that age-related barriers to social media use will diminish rapidly among future generations of cardiovascular disease (CVD) participants because of the rapid rise and increased use of smartphone and social media use among younger generations and the influence of younger family members on older people [8]. Moreover, encouraging older people to use social media and emphasizing the benefits of social media use would likely result in more engagement with and positive attitudes toward social media [27]. Older adults are still likely to use the Internet to seek health information and to connect to individuals experiencing similar conditions, mainly through discussion groups [11].

Social media–based discussion groups present an important new resource for health researchers to understand, given the primary role of social networks in the adoption of health behaviors [13,14]. Social media features such as information sharing allow social reinforcement to occur in real time [28,29]. However, the aforementioned barriers to social media use identified in this study may prevent online discussion groups from achieving peer-to-peer interactions, which are comparable to traditional in-person support groups. This may be overcome by supporting less capable users through peer-led mentoring, which is recognized as a potentially effective resource; however, it remains underinvestigated in chronic disease management [30]. Participants in this study, who were capable Facebook users, volunteered to act as mentors to less capable users to become more acquainted with Facebook. This approach was also suggested by Leist [16], who identified Internet-savvy members of online communities who were willing to act as mentors and at times, moderators. Such an approach has additional benefits, as it may promote continuous engagement of users in an online group, which is likely to maximize benefits [16].

Our study findings indicate that some participants are very capable in terms of engaging with personal interest groups via Facebook. However, none of the participants in this study recognized the potential Facebook provided for continued interaction with known cardiac experts and peers until prompted. Previous studies have reported that lack of personal relevance to a social media platform resulted in negative attitudes and lower engagement [20]. The use of well-known and existing social media platforms such as Facebook for interventions for less capable persons may be important for continued engagement [16]. Furthermore, the use of personally relevant social media platforms may have the potential to increase engagement in capable users, as found in this study—participants’ favored purposeful time spent on Facebook.

The majority of studies to date reporting social media interventions for health-related behavior change have documented outcomes rather than the process of intervention development. In 2014, a systematic review of studies, which incorporated Web-based social media platforms, reported that there is modest evidence that behavior change interventions targeting key modifiable health behaviors may be effective, but this review noted that this field of research is still in its infancy [21]. This review highlighted the importance of maximizing retention and engagement within social networking interventions and the limited information reported on the long-term effectiveness of the resultant behavior change. Of the 10 interventions included in the review [21], only five used an existing social media platform [31-35], with one using Twitter [34] and four using the private group feature of Facebook [31-33,35]. These four studies utilized private Facebook groups as an adjunct to an existing intervention to enhance social support for participants. None reported prior formative research on the social media experience and perspectives of their target group, and none of these studies involved participants with CVD.

The effectiveness of social media use as a delivery medium for the secondary prevention of cardiac rehabilitation remains unclear. Two systematic reviews investigating the effectiveness of mHealth interventions for the secondary prevention of CVD are available but are limited in that none of the included studies have investigator-built online social support elements or social networking via established social media platforms [15,36]. One digital cardiac rehabilitation intervention, designed as an adjunct to traditional in-person services, has been tested [37]. This intervention contained a Web-based application as well as a mobile phone app for personalized health assistance, coupled with a social reinforcement network that aimed to encourage the adoption and maintenance of a healthier lifestyle. However, no process evaluation data are available to understand how participants interacted with the social reinforcement network or the potential influence on outcomes. Furthermore, a study protocol describing a remotely delivered exercise-based cardiac rehabilitation mHealth program with a secure social support component has been designed and incorporates self-efficacy elements; however, it has not yet been implemented [38].

Insights of current Facebook use by participants attending cardiac rehabilitation in this study have provided an essential foundation for the design of a Facebook intervention for the secondary prevention of CVD. mHealth interventions, including those with social media elements, underpinned by behavioral theories have been shown to be more effective compared with mHealth interventions with no theoretical underpinning [39,40]. The themes from this study, namely, capability, opportunity, and motivation and interaction between them, have commonality with both social cognitive theory and self-efficacy [41] and with the behavior change wheel [42]. On the basis of evidence from interventions that are not based on social media, integrating principles from these behavior change theories for the secondary prevention of CVD may significantly increase the likelihood of success and therefore has potential for social media–based interventions. In addition, frameworks for adapting existing social media delivery will be taken into consideration [43], as well as the process of communicating health information via social media [44,45].

### Strengths and Limitations

The strength of this formative research is the use of both quantitative and qualitative data sources. A relatively diverse sample of both metropolitan and rural participants was represented in both phases of the study. However, it should be emphasized that the nature of the focus group study and qualitative research limits the generalizability of the results to the wider cardiac rehabilitation population, including those patients who are eligible for cardiac rehabilitation yet do not attend as well as the rural patients. It is possible that these subgroups could offer useful insights into accessibility of services; however, they are difficult populations to recruit and engage with.

### Conclusions

In this study, we have shown that group-based social media programs, using existing platforms such as Facebook, may offer an opportunity to access and engage cardiac rehabilitation patients. One in 4 cardiac rehabilitation patients are current users of Facebook—a proportion only likely to increase in the future, given the rapidly growing use of social media in older adults. Considering that cardiac rehabilitation patients who use social media are already frequently accessing information on their health, cardiac conditions, and lifestyle, it is important to leverage this potential with appropriate theory-based support. Capitalizing on the social media potential for cardiac patient support depends on understanding patients’ motivation to keep in touch with known health professionals and peers, efficiency of use **,** and ensuring that the benefits outweigh potential risks. This study has brought greater understanding of Facebook use and the perceptions of and engagement with Facebook by adults with CVD. As such, we have generated a set of recommendations for consideration when establishing cardiac rehabilitation Facebook groups. Future work will incorporate the findings and recommendations to develop a Facebook intervention to support adults with CVD to improve their modifiable risk factors and to lower their chances of further events.

## Abbreviations

CHD: coronary heart disease
CVD: cardiovascular disease
eHealth: electronic health
mHealth: mobile health
NSW: New South Wales
SD: standard deviation
SPSS: Statistical Package for the Social Sciences
